# Evolutionary Origin of Human *PALB2* Germline Pathogenic Variants

**DOI:** 10.3390/ijms241411343

**Published:** 2023-07-12

**Authors:** Jia Sheng Chian, Jiaheng Li, San Ming Wang

**Affiliations:** MoE Frontiers Science Center for Precision Oncology, Cancer Center and Institute of Translational Medicine, Faculty of Health Sciences, University of Macau, Macao; yb97618@um.edu.mo (J.S.C.); yb97645@um.edu.mo (J.L.)

**Keywords:** *PALB2*, evolutionary origin, phylogenetic, paleoanthropology

## Abstract

*PALB2* (Partner and localizer of BRCA2) is crucial for repairing DNA double-stranded breaks (DSBs) through homologous recombination (HR). Germline pathogenic variation in *PALB2* disrupts DNA damage repair and increases the risk of Fanconi Anemia, breast cancer, and ovarian cancer. Determination of the evolutionary origin of human *PALB2* variants will promote a deeper understanding of the biological basis of *PALB2* germline variation and its roles in human diseases. We tested the evolution origin for 1444 human *PALB2* germline variants, including 484 pathogenic and 960 benign variants. We performed a phylogenic analysis by tracing the variants in 100 vertebrates. However, we found no evidence to show that cross-species conservation was the origin of *PALB2* germline pathogenic variants, but it is indeed a rich source for *PALB2* germline benign variants. We performed a paleoanthropological analysis by tracing the variants in over 5000 ancient humans. We identified 50 pathogenic in 71 ancient humans dated from 32,895 to 689 before the present, of which 90.1% were dated within the recent 10,000 years. *PALB2* benign variants were also highly shared with ancient humans. Data from our study reveal that human *PALB2* pathogenic variants mostly arose in recent human history.

## 1. Introduction

*PALB2* is a tumor suppressor gene. It plays a key role in the repair of DNA double-stranded breaks (DSBs) through the DNA homologous recombination (HR) pathway [1]. *PALB2* functions as a central connecting hub to facilitate the formation of the BRCA1-PALB2-BRCA2 complex. The complex is essential for recruiting RAD51 for DNA strand exchange to repair at the sites of DSBs and also helps maintain the stability of stalled DNA replication forks [2]. Extensive studies showed that the carriers of monoallelic *PALB2* pathogenic variants (PVs) are predisposed to develop multiple types of cancer, including breast, pancreatic, and ovarian cancers [3,4,5]; the carriers of biallelic *PALB2* PVs can cause the subtype N of Fanconi anemia (FA-N) [6]. Furthermore, *PALB2* PVs contribute to BRCAness, the phenotype caused by the damaged homologous recombination (HR) function due to the defects in HR-involved non-BRCA1/2 genes [7,8]. BRCAness is the specific marker for cancer diagnosis, prevention, and treatment. The PARP inhibitors (PARPi) successfully used in the treatment of *BRCA*-deficient cancers through synthetic lethality can also be applied to treat the cancer types with *PALB2* pathogenic variation [9,10].

Knowledge for the origin of human *PALB2* germline PVs is important to understand the biological basis of *PALB2* germline PVs in cancer. However, little is known about the origin of human *PALB2* PVs although it is known that ChAM, a functional domain of *PALB2*, exhibits high evolutionary conservation [11], and human *PALB2* is under negative selection [12]. Our recent study revealed that the PVs in human *BRCA1* and *BRCA2* (*BRCA*) were not inherited through cross-species conservation but originated in recent human history [13]. These findings raise the question if the same patterns in human *BRCA* also exist in human *PALB2* germline PVs, considering the BRCAness features of human *PALB2* germline PVs.

In this study, we performed a phylogenetic analysis by searching human *PALB2* PVs in 100 non-human vertebrates. We did not find evidence to support evolutionary conservation as the source for human *PALB2* PVs. We further performed a paleoanthropological analysis by searching for human *PALB2* PVs in ancient humans. The results showed that human *PALB2* PVs mostly arose in the past 10,000 years. Therefore, our study demonstrated that both human *PALB2* PVs and human *BRCA* PVs arose in recent human history.

## 2. Results

### 2.1. Cross Species Conservation Analysis

From the Clinvar database, we identified a total of 1444 human *PALB2* germline variants consisting of 484 pathogenic variants (PVs) and 960 benign variants (BVs) for the study (Table S1). We downloaded from the UCSC genome browser the corresponding coordinates and bases of the 100 non-human vertebrates in 8 clades of Primates, Euarchontoglires, Laurasiatheria, Afrotheria, Mammals, Aves, Sarcopterygii and Fishs (Table S2). We searched the human *PALB2* germline variants in the 100 vertebrates to determine if there could be shared *PALB2* variants between humans and non-human species.

**Figure 1:**
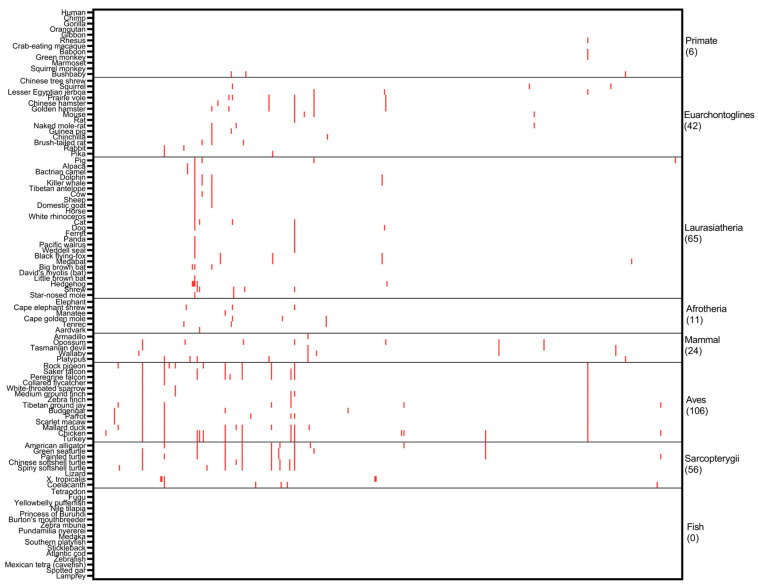
Cross-species analysis of *PALB2* germline PVs. Human *PALB2* PVs were searched in 100 non-human vertebrates. The results showed that cross-species conservation was unlikely the source of human *PALB2* germline PVs. The red cell represents the PVs shared between humans and other species. *X* axis: human *PALB2* PVs in Clinvar database; *Y* axis: 100 species from humans (**top**) in Primates to lamprey (**bottom**) in Fish. See Table S2A for details.

For *PALB2* PVs, we identified 81 (16.7%) human variants shared with 72 non-human species (Figure 1, Table S2A). Of the species sharing with human *PALB2* PVs, there were 21 species in Laurasiatheria clade of Mammalian class shared human c.2336C>G (p.Ser779Ter); chicken, mallard duck, and turkey of Aves clade had the sharing number of 15, 11 and 11, respectively. Species in Primates clade are close relatives of humans. However, only six human PVs were shared with the species in Primates, including three PVs of c.595del (pLeu199PhefsTer2), c.1451T>A (p.Leu484Ter) and c.1675C>T (p.Gln559Ter) shared with bushbaby, the most distal species in Primates to humans diverged 74 million years ago [14], and one PV of c.1018del (p.Glu347AsnfsTer9) shared between humans, rhesus, baboon and green monkey. Human PVs did not share with chimpanzees, gorillas, or orangutans. As the frequently used animal models in biomedical research, rat and mouse had only one and four *PALB2* PVs shared with humans. Zebrafish, another important model, did not share any PV with humans. Of the human PVs shared with multiple species, c.751C>T (p.Gln251Ter) was shared with 27 species, from prairie voles in Euarchontoglires to spiny softshell turtles in Sarcopterygii. We searched the sharing of two *PALB2* founder mutations of c.2323C>T (p.Gln775Ter) in the French Canadian population [15] and c.1592delT (p.Leu531Cysfs) in the Finnish population [16]. We found no evidence for their presence among the 100 species. As the majority of human *PALB2* germline PVs were not present in non-human species, and the small number of PVs shared between humans and other species did not follow the order of evolutionary tree, cross-species conservation was unlikely the source of human *PALB2* germline PVs.

**Figure 2:**
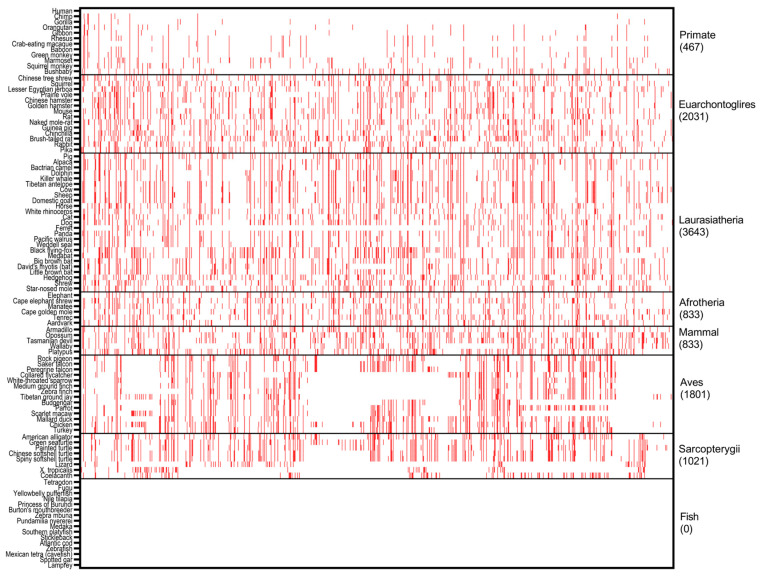
Cross-species analysis of *PALB2* germline BVs. The distribution of human *PALB2* BVs in 100 vertebrates is shown above. The red cell represents the BVs shared between humans and other species. *X* axis: human *PALB2* BVs in Clinvar database; *Y* axis: 100 species from human in Primates to lamprey in Fish. See Table S2B for details.

As a control, we performed the same analysis for human *PALB2* germline BVs. We identified 884 (90.5%) human variants shared with 83 species distributed in all clades except Fish (Figure 2, Table S2B). Platypus (194) and opossum (184) in Mammals and black flying fox (190) in Laurasiatheria had the highest sharing numbers. The most shared variant, c.3276C>G (p.Leu1092=), was distributed among 72 species from orangutan in Primates to coelacanth in Sarcopterygii. The sharing rate of *PALB2* BVs of Primates is higher than *PALB2* PVs, although it is still lower than other clades except Fish. For instance, c.2586+58C>T was shared by all the species within Primates, and Bushbaby in Primates had the highest sharing number of 86 BVs.

**Figure 3:**
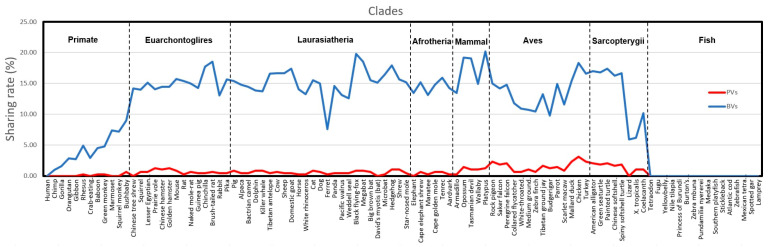
The sharing rates of human *PALB2* PVs and BVs with other non-human species. The sharing rates of each species were calculated by the number of shared variants divided by the total number of PVs or BVs. *X* axis: 100 species in the eight clades (Primates, Euarchontoglires, Laurasiatheria, Afrotheria, Mammals, Aves, Sarcopterygii, and Fish); *Y* axis: the sharing rate. See Table S2A,B for details.

The sharing patterns between *PALB2* PVs and BVs in 100 species were significantly different (*p* < 0.05) (Figure 3). The results indicated that evolutionary conservation was unlikely to contribute to the human *PALB2* germline PVs but a rich source for human *PALB2* germline BVs.

### 2.2. Ancient Human Genomic Analysis

**Figure 4:**
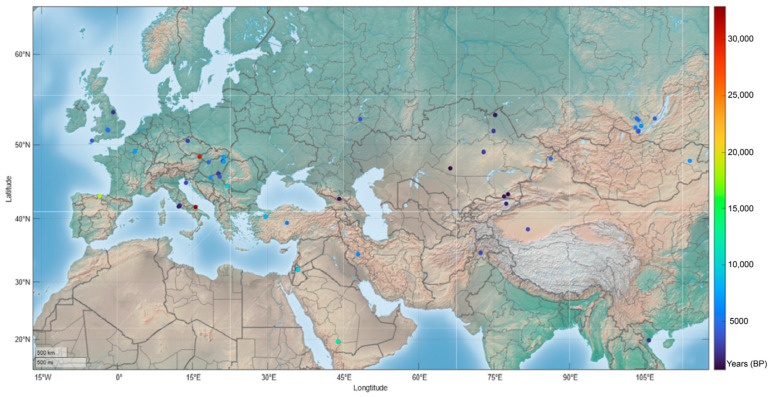
Geographic distribution of *PALB2* PVs identified in ancient humans. A total of 50 PVs was identified in 71 ancient humans dated from 32,895 to 689 BP; 64 (90.1%) PV carriers were mostly in Eurasia dated within the last 10,000 BP. Color gradient bar on right side shows the dated age of fossils. See Table 1 for details.

**Figure 5:**
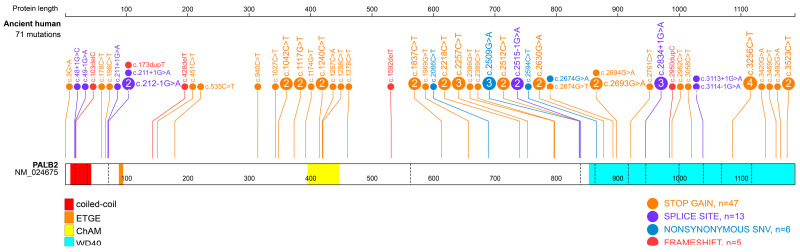
Distribution and mutation types of the 50 *PALB2* PVs identified in ancient humans. Each line represents the location of PV, and the frequencies of variants were shown in the numbers inside the circle if the PV had more than one carrier. See Table 1 for details.

As cross-species conservation is unlikely the source for human *PALB2* PVs, we then performed a paleoanthropological analysis to identify whether human *PALB2* PVs could be originated from human itself. We searched human *PALB2* PVs in the genomes of 5031 ancient human samples dated between 48,426 and 270 BP. We identified 50 human *PALB2* PVs in 71 ancient human carriers dated from 32,895 to 689 BP, of which 64 (90.1%) were dated within the recent 10,000 years before the present (BP) (Table 1, Figure 4). Stopgain variants constituted 64% (32/50) of the PVs identified in ancient humans, and 18 (36%) PVs were located at the WD40 domain in the C-terminus of PALB2 (Table 1, Figure 5). The oldest carrier sharing *PALB2* PV c.1837C>T (p.Gln613Ter) was an Italian dated about 32,985 BP, while the youngest carrier sharing *PALB2* PV c.49-1G>A was in Tian Shan, China, dated 689 BP. Of the 50 identified *PALB2* PVs, 16 (32%) were present in multiple ancient individuals. For instance, c.3256C>T (p.Arg1086Ter) was shared by four ancient individuals dated in 9860, 4475, 2175, and 1450 BP, accordingly; three *PALB2* PVs were shared by three ancient individuals, and seven *PALB2* PVs were shared by two individuals (Table 1). We did not identify the presence of the two reported *PALB2* founder mutations (c.2323C>T and c.1592delT) in ancient humans.

*PALB2* BVs were highly shared within ancient human populations. Of the 960 *PALB2* BVs, 234 (24.4%) were identified in 2141 ancient humans, of which 143 (61.1%) were synonymous SNV and 78 (33.3%) were intron variants, 107 (45.7%) were present in multiple individuals and 16 (6.8%) were shared with more than 10 individuals (Table S3). c.2996+264T>C had the highest sharing numbers of 553 carriers, and c.3114-51T>A were present in 370 carriers. A nonsynonymous SNV c.1676A>G (p.Gln559Arg) was shared in 62 individuals. The results indicated that human *PALB2* PVs arose during the recent human evolutionary process, whereas human *PALB2* BVs originated from both cross-species conservation and human evolutionary process.

## 3. Discussion

Our study investigated the evolutionary origin of human *PALB2* germline variation. Our comprehensive phylogenetical and paleoanthropological analyses demonstrate that *PALB2* germline PVs in modern humans were not originated through cross-species conservation but rather arose in the evolutionary process of human itself, likely within the past 10,000 years, if not earlier. We are confident for the 1st part of the conclusion that *PALB2* germline PVs in modern humans were not originated through cross-species conservation as the majority of human *PALB2* germline PVs were not present in non-human species, and the small number of PVs shared between human and other species did not follow the order of the evolutionary tree; we are also rather confident in the 2nd part of the conclusion that *PALB2* germline PVs in modern humans arose in human evolutionary process. However, whether *PALB2* germline PVs in modern humans fully originated in the past 10,000 years could be debatable by the fact that 4518 (89.8%) ancient humans available for the study were dated within the last 10,000 years, as it could bias the arising time limited within the 10,000 years. The possibility that the PVs could arise earlier cannot be ruled out unless they are absent in more ancient humans older than 10,000 years when available, which are currently not available.

By conducting phylogenetical analysis in 100 non-human vertebrate species, we found no evidence to support cross-species conservation as the source of *PALB2* PVs. In the species of Primates, only bushbaby, the most distant to human, shared three human PVs (c.595del, c.1451T>A and c.1675C>T). Rhesus, baboon, and green monkeys had only one PV c.1018del (p.Glu347AsnfsTer9) shared with humans. In contrast, the species in Aves and Sarcopterygii clades shared many human PVs. The sharing pattern may occur coincidentally rather than cross-species conservation, as it did not follow the evolutionary tree among the species. A proposed theory called “compensatory changes” proposed that the sharing of PVs between humans and distant species can be explained by other compensatory variants occurring at other coding or regulatory sites, neutralizing the deleterious effect of the mutations [36]. A deleterious variant in humans may be tolerated or have different impacts on other species. This may partially explain why the c.2336C>G (p.Ser779Ter) variant was only shared with 21 out of 25 species in the Laurasiatheria clade. Of the human PVs shared with multiple species, c.751C>T (p.Gln251Ter) was shared with 27 species, from prairie voles in Euarchontoglires to spiny softshell turtles in Sarcopterygii. The presence of human c.751C>T p.(Gln251Ter) in 27 species is likely by chance. Its presence only in 2/524 modern human families likely reflects its true prevalence in modern humans [5]. One of the possibilities exists that the C>T changes may also be de novo variants rather than originating from ancient humans. This is particularly true for the C>T variation at CpG islands, which is highly mutable due to high-degree spontaneous deamination [37,38]. In contrast, although the sharing rates of BVs in Primates were lower compared to other species, they followed the evolutionary relationship. Therefore, cross-species conservation can be a potential source for human *PALB2* BVs.

It is interesting that Fish clades did not share any PVs or BVs with humans. The evolutionary divergence between fish and human occurred approximately 450 million years ago [39]. Take zebrafish as an example: zebrafish PALB2 consists of only 459 amino acids, while human PALB2 has 1186 amino acids. Possible reasons for the absence of shared variants could be that the chromosome location of the zebrafish *Palb2* (chr1) is not the same as the human *PALB2* (chr16) and the vast differences in protein may prevent proper PALB2 alignment between fish and humans.

Of the 5031 ancient humans, 4518 (89.8%) were dated within 10,000 years. In the paleoanthropological analysis, we identified 71 carriers of 50 different *PALB2* PVs among ancient. Remarkably, 64 (90.1%) of these ancient carriers were dated within the past 10,000 years. This could be directly attributed to the significant human population expansion after the last glacial period around 10,000 years ago [40], the subsequent agricultural revolution [41], and the better preservation of ancient DNA for sequencing. 

The most common *PALB2* PV published is c.3113G>A p.(Trp1038Ter) (61/524 families), yet this did not appear in ancient humans, whereas the most frequent c.3256C>T p.(Arg1086Ter) in ancient humans was only present in 10/524 families [5]. This is an interesting feature in reflecting the dynamic prevalence of certain *PALB2* PVs along the human evolutionary process. For the PVs with severe deleterious impact, they can be suppressed by negative selection to prevent their spread in human population; for the PVs with both deleterious and “favorable” impacts on different developmental stages, they could be selected by positive selection. An example is the PVs in human *BRCA1*. While its classical function is DNA damage repair through HR and NHEJ pathways, human *BRCA1* gains multiple new functions, including regulation of immunity against viral infection [42], regulation of gene expression [43], regulation of neural development [44], and reproduction enhancement [45]. As such, human *BRCA1* is under strong positive selection [42] that leads to a high prevalence of 0.2–0.5% for *BRCA1* PVs, or one carrier in a few hundred individuals, in modern humans [46,47,48,49]. It is also important to note that most of the PVs arisen within a few thousand years ago were too young to be fixed in the genome, which can also lead to their unstable prevalence in either ancient or modern human populations. A possibility for c.3113G>A p.(Trp1038Ter) thought to be originated from the UK absent in ancient humans could be due to the small size of ancient samples and its lower prevalence that may not allow its detection, although 555 ancient humans from the UK were included in our study. It is also possible that c.3113G>A was young but has been rapidly spreading in the modern UK population.

Other factors contributing to the inconsistent prevalent rates of *PALB2* PVs in ancient and modern humans could be the different PV spectra used in the comparison. The study [50] included only the protein-truncating variants (PTVs) but other types of PVs were included in our study, a small sample size of ancient humans causing a punctured but not linear prevalence of certain PVs in ancient humans, poor quality and low coverage of DNA sequences of certain ancient humans, contributing to artifacts such as the false C>T variant caused by deamination, the time difference between ancient and modern human populations that the ancient humans had a wide distribution of over 30,000 years, whereas the PV-delivered modern humans had a narrow time window of few decades. Our results raise an interesting question whether the pathogenic variants in other DNA damage-repair genes would also follow a similar path as did for human *PALB2* PVs [51].

## 4. Methods and Materials 

### 4.1. Source of Human PALB2 Variants

The source of human *PALB2* variants was obtained from the ClinVar database (https://www.ncbi.nlm.nih.gov/clinvar/, accessed on 25 January 2023). The annotation of the *PALB2* variants utilized the following reference sequences: cDNA: NM_024675.4, protein: NP_078951.2, and genome (hg38): NC_000016.10. Only the single nucleotide variants (SNV) and indels involving a single base pair (bp) were included in the study. The study only included the pathogenic variants (PVs) and benign variants (BVs), as defined by existing pathogenicity classifications. PVs consisted of variants categorized as “Pathogenic” and “Likely pathogenic”, while BVs included variants classified as “Benign” and “Likely benign”. Variants with uncertain significance (VUS) and conflicting interpretations were excluded from the study.

### 4.2. Cross-Species Genomic Analysis

The *PALB2* sequences of 100 vertebrate species were obtained through a search on the UCSC genome browser (https://genome.ucsc.edu/, accessed on 15 March 2023). The detailed process of the phylogenetic analysis was described in our previous study [13]. In brief, the sequence alignment among these 100 species was performed following the steps outlined in Multiz Alignments within the UCSC genome browser [52]. The phyloP and PhyloFit programs were used to measure cross-species evolutionary conservation and construct a tree model based on the evolutionary relationships of the 100 vertebrate species [53,54]. The bases corresponding to human *PALB2* variants in the hg38 reference genome and other species were obtained using the GetBase tool [55]. LASTZ and Multiz were employed to align the sequences of the human genome and the non-human *PALB2* genome [56]. For indel alignments, any matched indel across all species was manually examined to ensure the accuracy of the alignment.

### 4.3. Ancient Human Genomic Analysis

Through searching PubMed and Allen Ancient DNA Resource (version 54.1) [57], we collected the genomic sequences of 5231 ancient human samples information and genome sequences dated from 48,426 to 270 BP. The ancient human genome data were downloaded, and the human genome reference sequences containing *PALB2* (chr16:23,603,165–23,641,310 in hg38 and chr16:23,614,486–23,652,631 in hg19) were used for mapping analysis. BWA algorithm was employed to map *PALB2* sequences to human genome sequences [58]. The resulting SAM files were converted to BAM files and sorted using SAMtools [59]. Duplicates were removed using MarkDuplicates, and the BAM files were further processed with the GATK toolkit using default settings for quality control, filtration, and variant calling [60,61]. The called ancient *PALB2* variants were annotated by ANNOVAR using default parameters to differentiate between known and novel variants and provide information about the variants [62]. The ancient human *PALB2* variants were then compared with those from the ClinVar database, which represents the *PALB2* variants in modern humans, to find the shared variants between modern and ancient humans. The estimated age and location of the ancient human fossils, which carried *PALB2* variants, were extracted from the original publications. For the *PALB2* PVs, the distribution and dated time for each variant were generated using MATLAB (The Math Works, Inc., Natick, MA, USA).

### 4.4. Statistical Analysis

Mann–Whitney U test was performed to compare the differences in the sharing rate of *PALB2* between PVs and BVs groups. The statistical analysis was performed using SPSS (version 26.0, IBM, New York, NY, USA). A *p*-value below 0.05 was considered statistically significant.

## Figures and Tables

**Table 1:** List of *PALB2* PVs identified in ancient humans.

Carrier Number	cDNA	Protein	Mutation Type	Domain	Arisen Time (BP) *	References
4	c.3256C>T	p.Arg1086Ter	stopgain	WD40	9860	[17,18,19]
3	c.2257C>T	p.Arg753Ter	stopgain	-	3325	[17,19,20]
3	c.2509G>A	p.Glu837Lys	nonsynonymous SNV	-	7050	[19,21,22]
3	c.2834+1G>A	-	splice site	WD40	6319	[23,24,25]
2	c.212-1G>A	-	splice site	ETGE	2275	[25,26]
2	c.211+1G>A	-	splice site	ETGE	10,050	[27]
2	c.1042C>T	p.Gln348Ter	stopgain	-	6319	[24,28]
2	c.1117G>T	p.Glu373Ter	stopgain	-	3157	[19,29]
2	c.1240C>T	p.Arg414Ter	stopgain	ChAM	3740	[23,30]
2	c.1837C>T	p.Gln613Ter	stopgain	-	32,895	[26,27]
2	c.2218C>T	p.Gln740Ter	stopgain	-	8315	[23,30]
2	c.2512C>T	p.Gln838Ter	stopgain	-	3871	[23,24]
2	c.2515-1G>A	-	splice site	-	4106	[17,24]
2	c.2630G>A	p.Trp877Ter	stopgain	WD40	5677	[17,31]
2	c.2693G>A	p.Trp898Ter	stopgain	WD40	>10,000	[24,32]
2	c.3523C>T	p.Gln1175Ter	stopgain	WD40	6713	[24]
1	c.3G>A	p.Met1Ile	startloss	-	31,630	[26]
1	c.48+1G>C	-	splice site	coiled-coil	30,260	[27]
1	c.49-1G>A	-	splice site	coiled-coil	689	[23]
1	c.103delC	p.Leu35PhefsTer18	frameshift deletion	coiled-coil	2758	[23]
1	c.173dupT	p.Leu58PhefsTer16	frameshift insertion	-	2554	[23]
1	c.178C>T	p.Gln60Ter	stopgain	-	6750	[22]
1	c.196C>T	p.Gln66Ter	stopgain	-	4158	[24]
1	c.428delT	p.Leu143ArgfsTer34	frameshift deletion	-	7178	[30]
1	c.451C>T	p.Gln151Ter	stopgain	-	8790	[22]
1	c.535C>T	p.Gln179Ter	stopgain	-	4885	[32]
1	c.940C>T	p.Gln314Ter	stopgain	-	1613	[23]
1	c.1027C>T	p.Gln343Ter	stopgain	-	2255	[25]
1	c.1174G>T	p.Glu392Ter	stopgain	-	4161	[23]
1	c.1257C>A	p.Cys419Ter	stopgain	ChAM	3900	[17]
1	c.1258C>T	p.Gln420Ter	stopgain	ChAM	725	[33]
1	c.1378C>T	p.Gln460Ter	stopgain	-	3440	[28]
1	c.1592delT	p.Leu531CysfsTer30	frameshift deletion	-	700	[23]
1	c.1969G>T	p.Glu657Ter	stopgain	-	2500	[34]
1	c.2066C>T	p.Ser689Leu	nonsynonymous SNV	-	6850	[18]
1	c.2386G>T	p.Gly796Ter	stopgain	-	1506	[19]
1	c.2389C>T	p.Gln797Ter	stopgain	-	4862	[26]
1	c.2594C>T	p.Ser865Leu	nonsynonymous SNV	WD40	4940	[31]
1	c.2674G>T	p.Glu892Ter	stopgain	WD40	8570	[35]
1	c.2674G>A	p.Glu892Lys	nonsynonymous SNV	WD40	3740	[30]
1	c.2694G>A	p.Trp898Ter	stopgain	WD40	>10,000	[32]
1	c.2761C>T	p.Gln921Ter	stopgain	WD40	2478	[23]
1	c.2950dupC	p.Leu984ProfsTer3	frameshift insertion	WD40	7221	[30]
1	c.2962C>T	p.Gln988Ter	stopgain	WD40	1300	[23]
1	c.3058C>T	p.Gln1020Ter	stopgain	WD40	1246	[23]
1	c.3114-1G>A	-	splice site	WD40	2279	[23]
1	c.3113+1G>A	-	splice site	WD40	1646	[26]
1	c.3420G>A	p.Trp1140Ter	stopgain	WD40	1246	[23]
1	c.3469C>T	p.Gln1157Ter	stopgain	WD40	8315	[30]
1	c.3492G>A	p.Trp1164Ter	stopgain	WD40	18,720	[27]

* Dated time of the oldest carriers (BP, before present).

## Data Availability

The data presented in this study are available in online Supplementary Materials.

## Supplementary Materials

The following supporting information can be downloaded at: https://www.mdpi.com/article/10.3390/ijms241411343/s1.
